# Crosstalk between VEGFR and other receptor tyrosine kinases for TKI therapy of metastatic renal cell carcinoma

**DOI:** 10.1186/s12935-018-0530-2

**Published:** 2018-03-05

**Authors:** Yongchang Lai, Zhijian Zhao, Tao Zeng, Xiongfa Liang, Dong Chen, Xiaolu Duan, Guohua Zeng, Wenqi Wu

**Affiliations:** Department of Urology, Minimally Invasive Surgery Center, Guangzhou Urology Research Institute, Guangdong Key Laboratory of Urology, The First Affiliated Hospital of Guangzhou Medical University, Kangda Road 1#, Haizhu District, Guangzhou, 510230 Guangdong China

**Keywords:** TKIs, Crosstalk, mRCC, VEGFR, HIFs, RTKs, Targeted therapy

## Abstract

Clear cell renal cell carcinoma (ccRCC) is the most common subtype of renal cell carcinoma (RCC), and is frequently accompanied by the genetic features of von Hippel–Lindau (VHL) loss. VHL loss increases the expression of hypoxia-inducible factors (HIFs) and their targets, including epidermal growth factor (EGF), vascular endothelial growth factor (VEGF), and platelet-derived growth factor (PDGF). The primary treatment for metastatic RCC (mRCC) is molecular-targeted therapy, especially anti-angiogenic therapy. VEGF monoclonal antibodies and VEGF receptor (VEGFR) tyrosine kinase inhibitors (TKIs) are the main drugs used in anti-angiogenic therapy. However, crosstalk between VEGFR and other tyrosine kinase or downstream pathways produce resistance to TKI treatment, and the multi-target inhibitors, HIF inhibitors or combination strategies are promising strategies for mRCC. HIFs are upstream of the crosstalk between the growth factors, and these factors may regulate the expression of VEGR, EGF, PDGF and other growth factors. The frequent VHL loss in ccRCC increases HIF expression, and HIFs may be an ideal candidate to overcome the TKI resistance. The combination of HIF inhibitors and immune checkpoint inhibitors is also anticipated. Various clinical trials of programmed cell death protein 1 inhibitors are planned. The present study reviews the effects of current and potential TKIs on mRCC, with a focus on VEGF/VEGFR and other targets for mRCC therapy.

## Background

Renal cell carcinoma (RCC) is the most common kidney solid neoplasm, and 12 drugs are approved in US for metastatic RCC (mRCC). RCC is distinguished into three major histopathological classifications: clear cell RCC (ccRCC; 70–75%), papillary RCC (pRCC; 10–16%), and chromophobe RCC (chRCC; 5%) [1]. Approximately 60–80% of ccRCC cases exhibit the most frequent genetic feature, the loss of von Hippel–Lindau (VHL) [2, 3], which increases the expression of hypoxia-inducible factors (HIFs), their targets, and cell survival [4, 5]. HIF-2 is implicated in angiogenesis, and some ccRCCs are HIF-2 independent [6], which triggered biomarker-driven clinical trials. Biomarkers to predict outcome using targeted therapy in metastatic ccRCC exhibited some promise but further validation is needed [7–11]. Patients confronted with rare kidney cancers are often treated in the same manner as ccRCC patients [12]. The prognosis of mRCC is poor and the primary treatment is molecular-targeted therapy. Targeted therapy developed quickly and tyrosine kinase inhibitors (TKIs), mammalian target of rapamycin (mTOR) inhibitors and the programmed cell death protein 1 (PD-1)/programmed death ligand 1 (PD-L1) checkpoint inhibitors (such as nivolumab) are the standard target therapies for mRCC [13–15].

**Fig. 1:**
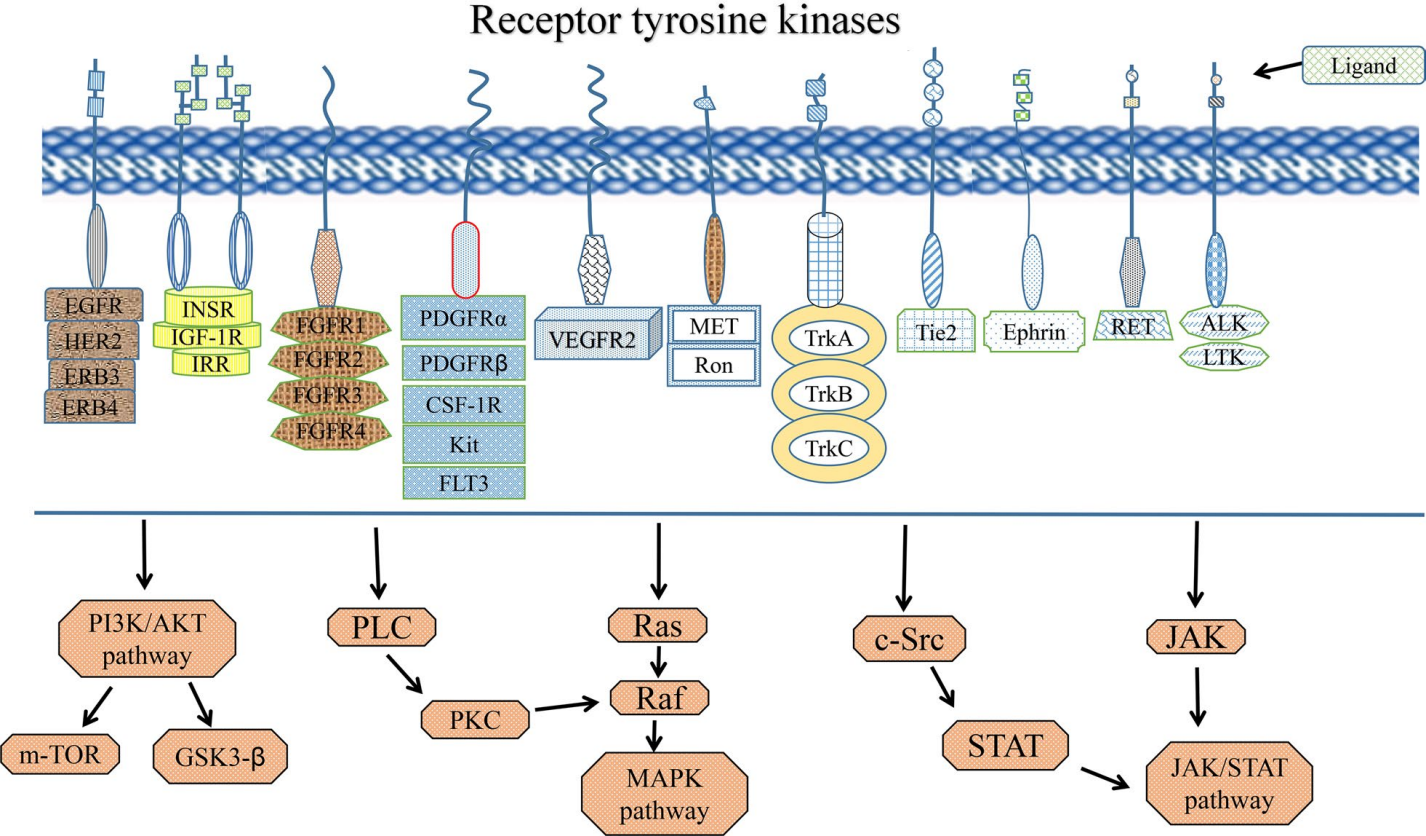
Receptor tyrosine kinases, including EGFR, VEGFR, FGFR, PDGFR, and IGF-1R, are shown. Activation of tyrosine kinases initiates multiple downstream signalling pathways, including PI3K/AKT, MAPK, and JAK/STAT pathways and so on, which become the basis of the crosstalk between TKs

Receptor tyrosine kinases (RTKs), include epidermal growth factor receptor (EGFR), vascular endothelial growth factor receptor (VEGFR), fibroblast growth factor receptor (FGFR), platelet-derived growth factor receptor (PDGFR), and insulin-like growth factor 1 receptor (IGF-1R). Activation of tyrosine kinases (TKs) initiates multiple downstream signalling pathways, including phosphatidylinositol 3-kinase (PI3K)/AKT, Ras/Raf/MEK/ERK1/2, phospholipase C (PLC), signal transducer and activator of transcription (STAT)3 and STAT5 pathways [16, 17]. These multiple downstream signalling pathways are the basis of the crosstalk between TKs (Fig. 1).

Twelve TKs (e.g., ABL2, CSF1R, and MET) are significantly upregulated in ccRCC, and 7 TKs (e.g., ERBB4, PDGFRA, ERBB2, and FGFR3) are downregulated [18]. Selective TKIs exhibited promise in the treatment of cancers driven by activated TKs. For example, TKIs for direct to Bcr-Abl, c-Kit and EGFR exhibited promise in the treatment of chronic myelogenous leukaemia, stromal tumours, and non-smallcell lung cancer (NSCLC) respectively. Numerous monoclonal antibodies directed against receptors or ligands and TKIs, such as cabozantinib [19], XMD8-87 (ACK inhibitor) [20] and axitinib [21, 22], were developed or approved (Table 1).

**Table 1:** Ligands and inhibitors of protein tyrosine kinases

Protein tyrosine kinase	Ligand	Monoclonal antibody of ligand	Representative TKI
VEGFR	VEGF (A, -B, -C, -D, -E)	Bevacizumab, aflibercept, ramucirumab (anti-VEGFR2)	Sorafenib, sunitinib, axitinib, pazopanib
EGFR	EGF, TGFα, HB-EGF, amphiregulin, epiregulin, epigen, β-cellulin, NRG 2 β	Nimotuzumab, panitumumab, cetuximab, necitumumab (anti-EGFR)	Erlotinib, afatinib, osimertinib, sapitinib
PDGFR	PDGF	Olaratumab (anti-PDGFRα)	Imatinib, pazopanib
c-MET (HGFR)	HGF		Cabozantinib [19], crizotinib
HER2		Trastuzumab,ramucirumab, pertuzumab	Lapatinib, sapitinib
IGF-1R	IGF-1		Linsitinib, GSK1904529A
FGFR	FGF		Nintedanib, NVP-BGJ398
FLT3	FLT3 ligand		Quizartinib, dovitinib
c-Kit	Stem cell factor		Dovitinib, pazopanib
Tie-2	Angiopoietin		Pexmetinib
c-RET	GDNF, neurturin, artemin, persephin		Regorafenib
TAM receptor	Gas6, protein S		Sitravatinib
CSF-1R	CSF-1		Linifanib
Ephrin receptor	Ephrins		Sitravatinib
Trk receptor	BDNF, NGF		Sitravatinib, larotrectinib
ACK			XMD8-87 [20]
Src			Bosutinib
ALK			Crizotinib

## VEGF/VEGFR downstream pathway and VEGFR-TKI

VEGF family members in mammals consist of VEGF-A, -B, -C, -D, -E and placenta growth factor (PLGF). There are three main isoforms of VEGFR, VEGFR-1, VEGFR-2 and VEGFR-3, and VEGFR-2 plays a key role in angiogenesis [23]. VEGFR-3 is primarily expressed on lymphatic vessels, but the other VEGFR and the Tie receptor family are primarily expressed specifically in the endothelium. VEGF-A stimulates VEGFR2, which is autophosphorylated and activates various downstream signaling pathways [24]. Anti-angiogenesis, especially VEGF/VEGFR targeted theraphy, emerged as the standard of care for mRCC. Numerous VEGFR**-**TKIs were designed and developed (Table 2). VEGFR2-TKIs, such as sorafenib or sunitinib, are valuable treatment approaches for patients with mRCC [25]. VEGF and VEGFR polymorphisms affected outcomes in sunitinib-treated mRCC patients, especially VEGFR1 polymorphisms [26].

**Table 2:** Familiar VEGFR tyrosine kinase inhibitors and their targets

TKI	VEGFR-1	VEGFR-2	VEGFR-3	Other targets
Sorafenib		+		Raf-1, B-Raf, B-Raf (V599E)
Sunitinib		+		c-Kit, FLT3, PDGFRβ
Lenvatinib	+	+	+	PDGFRα, PDGFRβ, FGFR1
Cabozantinib [19]		+		c-MET, AXL, RET, KIT, FLT3, TRKB, Tie-2
Axitinib [21, 22]	+	+	+	PDGFRα, PDGFRβ, Kit, BCR-ABL1
Vandetanib		+	+	EGFR
Dovitinib	+	+	+	c-Kit, FLT3, FGFR1
Pazopanib	+	+	+	PDGFR, FGFR, c-Kit
Foretinib	+	+	+	MET, Tie2
Apatinib		+		RET

### Mechanisms of TKI resistance

TKIs treatments are associated with dynamic changes in relevant biomarkers, including other protein TKs [27]. For example, VEGFR-TKI treatment significantly reduced vessel density (CD31) and phospho-S6K, PD-L1, and FOXP3 expression and significantly increased Ki-67, cytoplasmic FGF-2 and MET receptor expression in vessels [27]. However, long-lasting efficacy is seldom achieved, and evasive resistance eventually occurs under anti-angiogenic TKI therapy [28]. A recent study suggested that long non-coding RNAs (lncRNAs) play a significant role in TKI resistance in RCC. lncRNA-SRLR may be resistant to sorafenib and serve as a predictive biomarker for sorafenib tolerance via directly binding to NF-κB and the promotion of IL-6 transcription, which leads to STAT3 activation [29].

Exosomes also play a key role in resistance to drug theraphy. Stromal cells orchestrate an intricate crosstalk with tumour cells via utilization of exosomes to expand therapy resistance and reinitiate tumour growth [30]. lncARSR may promote and disseminate sunitinib resistance via competitively binding to miR-34/miR-449 to facilitate AXL and c-MET expression, incorporating into exosomes and transmitting to sensitive in RCC cells [31]. MiR-21 and miR-126 are targets of lncRNAs, and these molecules may be probable prognostic markers and therapeutic targets in RCC [32].

Various multi-targeted TKIs were developed because resistance to TKI treatment is inevitable. Sorafenib is a multi-targeted TKI that significantly improved clinical outcomes of mRCC patients [33]. However, no significant differences between sorafenib and sunitinib were observed in the treatment of advanced renal cancer in Chinese patients [34]. Axitinib, bevacizumab, and pazopanib are also anti-angiogenic drugs that approved for use in mRCC. Interleukin (IL)-8 expression is elevated, during sunitinib resistance, which suggests that IL-8 is also an key contributor and a potential therapeutic target to reverse sunitinib resistance in ccRCC [35]. Patients with high concentrations of IL-8, osteopontin and HGF exhibited shorter progression-free survival (PFS) compared to patients with low IL-8 concentrations using pazopanib as a treatment drug [36]. Cabozantinib is an important new standard-of-care treatment option for patients with advanced RCC who previously received anti-angiogenic therapy [37, 38]. VEGF is the downstream target of the HIF signal, and drugs that inhibit HIF-2 are in various stages of clinical testing [5]. The targeting of angiogenesis and hypoxia pathways may provide a resolution for the anti-angiogenesis resistance [28]. The HIF2α antagonist PT2385 is a novel therapeutics for RCC, and it exhibited cogent preclinical efficacy and improved tolerability [39]. Table 3 shows the factors, genes, proteins and other molecules (e.g., P-gp, MRP, and GSTs) involved in TKI resistance.

**Table 3:** Factors, genes or proteins involved in TKI resistance

Resistance type	Factors	Genes	Proteins
Intrinsic resistance	High glucose uptake	Tumour suppressor gene loss, polymorphism or mutation, such as VHL, TP53, PTEN, EGFR T790M and so on	TP53, BIM, HIF, P-gp, MDR1, GSTs, MRP and so on
Acquired resistance	Exosomes; lncRNA-SRLR and lncRNA-ARSR; miRNA 451, 221, 30a and so on [90]; EMT	Crosstalk, bypass and downstream signal activation or amplification (such as PI3K/AKT pathway)	IL-8, VEGFR-3, KRAS, BRAF, PDGFR, EGFR, FGFR, c-MET, AXL and so on

### Side effects of TKIs

The development of TKIs is revolutionary progress, but TKIs exhibit side effects, including cardiovascular side effects, especially hypertension and congestive heart failure, and continual clinical monitoring should be emphasized in the use of new TKI agents [40, 41]. Cardiac damage from TKIs (sorafenib and sunitinib) treatment is a largely underrated phenomenon, but it is manageable with careful cardiovascular monitoring and cardiac treatment at the first signs of myocardial damage [42]. Another TKIs, cabozantinib, also exhibited a manageable adverse events profile in patients with advanced RCC [37]. Sunitinib increases buccodental toxicity compared to chemotherapy [43].

### TKIs and immunotherapy

Immunotherapy enjoyed tremendous development recently in the form of immune checkpoint inhibition and vaccines [44]. VEGF-A/VEGFR-2 is also related to with tumour escape. VEGF-A directly triggers Treg proliferation, and VEGF-A/VEGFR-2 blockade inhibits this effect. Therefore, anti-VEGF-A therapies may also exert immunological effects [45]. A combination of immunotherapy treatment is also in process [46]. The combination of an IL-6 inhibitors (tocilizumab) and TKIs (sorafenib) may be a novel therapeutic approach for RCC [47]. Anti-VEGF (bevacizumab) in combination with an anti-PD-L1 (atezolizumab) improved antigen-specific T cell migration in mRCC [48]. More drug combination experiments will be performed with the design and development of less toxic novel immune checkpoint inhibitors and TKIs.

## VEGFR-TKIs and other signalling pathways

Molecular crosstalk between VEGFR and other TKs or downstream pathways, such as EGFR, c-Met, FGFR, PDGFR, IGF-1R, c-Kit and PI3K/AKT/mTOR, may have great therapeutic and resistance implications [23, 49]. The crosstalk between these factors contributes to TKI resistance, but multi-targets or combination drugs may exhibit good synergy. Therefore, various multi-target inhibitors were examined or in process and some of these are listed below.

### The mTOR pathway and its inhibitors

The mTOR/Raptor complex (mTORC1) is a key molecule in the PI3K/AKT/mTOR signalling pathway, and its activation increases protein synthesis and cell survival via direct phosphorylation of its effectors. Inhibitors of mTOR, such as everolimus and temsirolimus, are approved for the treatment of mRCC. Administration of everolimus alone or with lenvatinib is one of the most effective options for mRCC [50]. However, the inhibition of mTORC1 produces a loss of negative feedback loops, which upregulates the downstream effectors of the PI3K/AKT/mTOR pathway and activates of HIFs (an activator of angiogenesis) [51].

A combination of agents targeting the multiple pathways of angiogenesis, including HIF, VEGFR, PI3K and mTORC1/2, will likely be a beneficial choice. Lenvatinib plus everolimus and lenvatinib alone improved PFS in patients with mRCC who progressed after administration of one previous VEGF-targeted therapy [52]. The combination of bevacizumab and temsirolimus in patients previously treated with VEGFR-TKI is possible, but with dose reductions and treatment discontinuations [53]. The antidiabetic drug metformin blocks cell growth via TORC1 inhibition, and the combination of metformin and VEGF-TKI may be effective [54]. Combined treatment with everolimus and a Toll-like receptor 9 agonist immune modulatory oligonucleotide effectively interfered with tumour growth and angiogenesis in VHL wild-type and mutant models of RCC [55].

### VEGFR and other tyrosine kinase pathway or TKIs

#### EGF/EGFR pathway and TKIs

EGF family ligands include EGF, heparin-binding EGF-like growth factor (HB-EGF), transforming growth factor alpha (TGFα), amphiregulin, epiregulin, epigen, β-cellulin, and neuregulin 2β (NRG2β) [56]. The human EGFR family consists of EGFR (ERBB1), HER2 (ERBB2), HER3 (ERBB3), and HER4 (ERBB4) [57]. Activation of HER2 and EGFR activates intracellular pathways, such as RAS/RAF/MEK/ERK, PI3K/AKT/TOR, Src kinases, and STAT transcription factors. The EGFR gene is upregulated in ccRCC [58], and the HIF may activate the TGF-α/EGFR pathway to promote the growth of VHL(−/−) RCC cells [59].

Three generations of EGFR-TKIs were developed. The first generation of EGFR-TKIs such as erlotinib or gefitinib, exhibit resistance after several months of treatment in patients with EGFR-activating mutations, especially in NSCLC patients [60]. The EGFR T790M mutation confers resistance to gefitinib via blockade of drug binding [61]. Therefore, the second generation of EGFR-TKIs such as afatinib and dacomitinib, were developed. However, the expression of FGFR1 and its ligand FGF2 is enhanced in afatinib-resistant cancer cells, which provide an escape mechanism for cell survival [62]. Second-generation drugs inhibit EGFR T790M, but these agents also inhibit wild-type EGFR. Therefore the dose-limiting toxicities from wild-type EGFR inhibition prevent the administration of doses that are sufficient to fully suppress T790M. The third generation of EGFR-TKIs, such as osimertinib, were developed to overcome these limitations [63].

Compensatory TK signalling is observed in EGFR-TKI therapy, and KRAS, anaplastic lymphoma kinase (ALK), c-MET and BRAF mutations are also associated with poor responses to anti-EGFR therapy in some cancers. Adaptation to TKI treatment also reactivates ERK signalling in TK-driven malignancies [64]. A novel dual inhibitor of EGFR and c-MET, TC-N19, was investigated as a potential new-generation TKI inhibitor to treat resistance to current TKI-targeting therapies [65]. Resistance to EGFR-targeted agents may also be related to increased VEGF levels. Vandetanib, is an inhibitor of EGFR, VEGFR and RET TK that exhibited therapeutic efficacy, and it received FDA approval for the treatment of advanced medullary thyroid carcinoma [66].

AEE788 is another potent inhibitor of EGFR and VEGFR TKs at the isolated enzyme level and in cellular systems [67], AEE788 profoundly reduce RCC cells growth in vitro [68]. However, the VEGF/VEGFR signal is the primary target because the universal VHL loss in ccRCC, and the mTORC1, MET and IL–8, but not the EGFR or PI3K pathways are secondary targets based on the available clinical and preclinical studies in mRCC [4].

#### PDGF/PDGFR and TKIs

The PDGF family consists of PDGF-A to -D polypeptide homodimers and the PDGF-AB heterodimer, and these ligands can bind to PDGFR-α and -β tyrosine kinase receptors [23]. The HIF signalling pathway regulates the target genes VEGF, EGF, TGF-β, and PDGF. High expression of PDGFR-β and α-smooth muscle actin (α-SMA) and low vessel density were significantly associated with short survival in RCC [69]. Other PTK pathways, such as PDGF/PDGFR and FGF/FGFR pathways, provide underlying escape mechanisms from anti-VEGF/VEGFR therapy that may promote resumption of tumor growth [23]. Multiple inhibitors, such as sunitinib, pazopanib, axitinib, tivozanib, linifanib, telatinib and motesanib, that inhibit VEGFR and PDGFR TKs have been used [70].

#### HGF/c-MET and TKIs

Met and its ligand, hepatocyte growth factor (HGF), play significant roles in multiple oncogenic cellular processes, including regulation of cell proliferation, invasion, angiogenesis and alternative pathways to the VEGF [49]. MET mutations are frequently found in Papillary RCC (pRCC) [71]. PD-L1 and PD-L2 in ccRCC is associated with adverse features of c-MET and VEGF expression, respectively [72].

The role for Met in resistance to other RTK-targeted therapies is associated with crosstalk between Met and other receptors, such as EGFR, HER2 and VEGFR [49]. Combination targeting of the VEGF and c-MET pathways in a ccRCC model exhibited a better anti-tumour effect than single agent administration [73]. Cabozantinib is a TKI inhibitor of VEGFR, c-MET and other TKs that exhibited significant clinical benefit in PFS and objective response rate over the standard-of-care sunitinib as first-line therapy in patients with intermediate- or poor-risk mRCC [74].

#### IGF system pathway

The insulin-like growth factor (IGF) system is comprised of multiple growth factor receptors, including IGF-1R, insulin receptor (IR)-A and -B [75]. IR is primarily expressed in adipose tissue, the muscle and liver in adult tissues, and IGF-1R is expressed in most human tissues [76]. As a TK receptor for IGF-1 and IGF-2, IGF-1R plays a key role in proliferation, malignant transformation, anti-apoptosis and metastasis. IGF-1R expression in RCC is associated with poor long-term patient survival [77]. The risk of death for patients with IGF-1R overexpression increases 70% compared to ccRCC patients with tumours without IGF-1R expression [78].

IGF-1 co-culture with cells facilitates angiogenesis via the PI3K/Akt signalling pathway [79]. IGF-1R also exhibits crosstalk with the VEGF/VEGFR, EGF/EGFR pathway [80, 81]. IGF-1R also confers resistance to EGFR or VEGFR family targeted therapies [75]. A bi-functional antibody-receptor domain fusion protein that targeted IGF-IR and VEGF for degradation, bi-AbCap, exhibited superior inhibition of tumour growth in RCC, colon cancer, and pancreatic cancer compared to a combination of anti-IGF-IR and anti-VEGF therapies [82].

#### Other TKIs

Table 1 shows other RTKs, such as FGFR (FGFR1-4), tyrosine receptor kinase (Trk), ephrin receptor, ALK and Src. FGF/FGFR regulates normal and tumour cells growth, differentiation and angiogenesis, and the complex interaction and crosstalk between tumour angiogenic factors, such as FGF2 and PDGFR, promoted tumour growth and metastasis [83].

TrkA and TrkB are neurotrophin receptors. TrkB activation or overexpression could promotes proliferation, survival, angiogenesis, anoikis-resistance and metastasis in tumours. Brain-derived neurotrophic factor binds to TrkB and p75NTR and induces cell survival and migration via p75NTR, which is independent of TrkB activation [84], which indicates a resistance mechanism of TKIs for TrkB. TrkB silencing improved the anticancer efficiency of sorafenib in anoikis-resistant ACHN (a renal cancer cell line derived from metastatic site) RCC cells via inactivation of PI3K/Akt and MEK/ERK pathways [85].

Many other non-receptor TKs exist and numerous TKIs are under investigation.

## Conclusions

System treatment using multi-target drugs, immune checkpoint inhibitors or drugs combinations may be a promising approach to RCC therapy in the future because of the emergence of drug resistance to VEGFR-TKI, which may facilitate tumour invasiveness and metastasis. Three new second-line treatments received FDA approval in the last year for use after anti-angiogenic therapy: nivolumab, cabozantinib, and the combination of the TKI lenvatinib and everolimus (the mTOR inhibitor) [86]. Nivolumab is an immune checkpoint inhibitor, and cabozantinib is a multi-target TKI. The potential synergistic activity of antiangiogenic agents and TKIs or immunotherapy in NSCLC was summarized previously [87].

Combinations of VEGFR-TKIs, immune checkpoint inhibitors, and HIF inhibitors may provide good options to overcome drug resistance. Increasing evidence supports a primary role for the HIF-2α subunit in ccRCC over HIF-1α. Due to the frequently VHL loss in ccRCC, which causes increased expression of HIFs, targeting HIFs may be a promising strategy for ccRCC because of frequent VHL loss, which increases HIF expression. HIFs are upstream of the crosstalk between growth factors and regulate the expression of VEGR, EGF, PDGF and other growth factors, which supports targeting HIFs to overcome TKI resistance. Recent preclinical and clinical data on ccRCC indicate that PT2385 and PT2399 effectively inhibit cancer cell growth, proliferation, and tumour angiogenesis characteristic [88, 89]. The combination of HIF and immune checkpoint inhibitors is also anticipated, and various clinical trials for PD-1 inhibitors are planned.

## Abbreviations

RCC: renal cell carcinoma
ccRCC: clear cell renal cell carcinoma
mRCC: metastatic renal cell carcinoma
VHL: von Hippel–Lindau
HIFs: hypoxia-inducible factors
EGF: epidermal growth factor
VEGF: vascular endothelial growth factor
PDGF: platelet-derived growth factor
TKIs: tyrosine kinase inhibitors
mTOR: mammalian target of rapamycin
PD-1: programmed cell death protein 1
PD-L1: programmed death ligand 1
EGFR: epidermal growth factor receptor
VEGFR: vascular endothelial growth factor receptor
FGFR: fibroblast growth factor receptor
PDGFR: platelet-derived growth factor receptor
IGF-1R: insulin-like growth factor 1 receptor
TK: tyrosine kinase
RTK: receptor tyrosine kinase
PI3K: phosphatidylinositol 3-kinase
PLC: phospholipase C
STAT: the signal transducer and activator of transcription
NSCLC: non-smallcell lung cancer
lncRNA: long non-coding RNA
IL: interleukin
mTORC1: mTOR/raptor complex
PFS: progression-free survival
HB-EGF: heparin-binding EGF-like growth factor
TGFα: transforming growth factor alpha
α-SMA: α-smooth muscle actin
EMT: epithelial to mesenchymal transformation
BIM: Bcl-2 interacting mediator of cell death
P-gp: P-glycoprotein
MDR1: multidrug resistance protein 1
GSTs: glutathione *S*-transferase
MRP: multidrug resistance-associated protein
CSF-1: colony stimulating factor 1
FLT3: Fms-like tyrosine kinase 3
BDNF: brain-derived neurotrophic factor
GDNF: glial cell line-derived neurotrophic factor
NGF: nerve growth factor
NRG2β: neuregulin 2β
Trk: tyrosine receptor kinase
HGF: hepatocyte growth factor
ALK: anaplastic lymphoma kinase
